# Combined Use of Polyurethane Prepolymer and Aromatic Oil in Physicochemical Rejuvenation of Aged SBS Modified Bitumen for Performance Recovery

**DOI:** 10.3390/polym15051120

**Published:** 2023-02-23

**Authors:** Suxun Shu, Guofu Chen, Jiaming Yan, Ziqing Li, Weili Shen, Kai Gong, Yi Luo

**Affiliations:** 1 School of Civil Engineering and Architecture, Wuhan Institute of Technology, Wuhan 430073, China; 2 Hubei Provincial Engineering Research Center for Green Civil Engineering Materials and Structures, Wuhan 430074, China

**Keywords:** aged SBSmB, reaction rejuvenation, structural reconstruction, polyurethane, aromatic oil

## Abstract

The high-quality reutilization of waste styrene–butadiene–styrene copolymer (SBS) modified asphalt mixtures is a difficult issue in the field of highways today, and the main reason is that conventional rejuvenation technology fails to achieve the effective rejuvenation of aged SBS in binder, causing significant deterioration in the high-temperature performance of the rejuvenated mixture. In view of this, this study proposed a physicochemical rejuvenation process using a reactive single-component polyurethane (PU) prepolymer as the repairing substance for structural reconstruction and aromatic oil (AO) as a common rejuvenator used to supplement the lost light fractions of asphalt molecules in aged SBSmB, according to the characteristics of oxidative degradation products of SBS. The joint rejuvenation of aged SBS modified bitumen (aSBSmB) by PU and AO was investigated based on Fourier transform infrared Spectroscopy, Brookfield rotational viscosity, linear amplitude sweep, and dynamic shear rheometer tests. The results show that 3 wt% PU can completely react with the oxidation degradation products of SBS and rebuild its structure, while AO mainly acted as an inert component to increase the content of aromatic components, thereby reasonably adjusting the compatibility of chemical components of aSBSmB. Compared with the PU reaction-rejuvenated binder, the 3 wt% PU/10 wt% AO rejuvenated binder had a lower high-temperature viscosity for better workability. The chemical reaction between PU and SBS degradation products dominated in the high-temperature stability of rejuvenated SBSmB and had a negative impact on its fatigue resistance, while the joint rejuvenation of 3 wt% PU and 10 wt% AO not only gave a better high-temperature property to aged SBSmB but could also have the capacity to improve its fatigue resistance. Compared to virgin SBSmB, PU/AO rejuvenated SBSmB has comparative low-temperature viscoelastic behavior characteristics and a much better resistance to medium-high-temperature elastic deformation.

## 1. Introduction

With the continuous development of highway construction, asphalt pavement has become the mainstream road surface in China due to its advantages of comfortable driving, low noise, and easy maintenance [1,2,3]. As ordinary petroleum asphalt pavement is prone to aging in the natural environment, it is prone to ruts, pits, and other damage under the repeated action of different traffic loads and is destroyed before reaching the design life [4,5,6]. In order to prolong the service life of ordinary asphalt pavement, the polymer styrene–butadiene–styrene (SBS) has been used on a large scale to prepare modified bitumen in expressways and other high-grade roads in China in the past 20 years, so as to improve the overall performance of asphalt pavement, especially resistance to deformation at high temperature and cracking at low temperature [7,8,9].

It is well recognized that the addition of modifiers such as SBS into virgin bitumen can improve its high-temperature rutting resistance, low-temperature cracking resistance, pavement fatigue resistance and good elasticity and toughness [10,11,12,13]. As with ordinary bitumen, SBS modified bitumen (SBSmB) will undergo oxidative aging due to the comprehensive effects of sunlight, geothermal, air and rain erosion during service, which will lead to structural damage to the pavement, requiring renovation or reconstruction, resulting in a large quantity of waste SBSmB concrete [14,15,16,17]. Although SBS can significantly improve the high- and low-temperature properties of base bitumen, with the extension of service time, some SBS modified asphalt pavement constructed earlier have been seriously damaged, and a large quantity of waste SBSmB mixture will be produced every year. Disposal of these excavated or milled SBSmB materials will not only cause serious pollution to the ecological environment but will also cause great waste of these non-renewable resources [18,19,20]. Although it is common practice in some countries, such as Italy, to recycle asphalt mixtures containing SBSmB for warm mix asphalt pavement, it still cannot completely solve the above problems [21,22]. Therefore, it is of great practical significance to carry out high-quality recycling of waste SBSmB mixtures.

At present, some progress has been made in research on the rejuvenation of aged SBSmB. Yan et al. [23] studied the influence of tung oil on the recycling effect of aged SBSmB and concluded that there was no chemical interaction between tung oil and aged SBS modified bitumen, which could obviously improve the low-temperature crack resistance and flexibility of aged SBSmB, and the high-temperature performance of rejuvenated SBSmB could be better guaranteed only when its content was effectively controlled. Cao et al. [24] selected four rejuvenators with different chemical compositions (vacuum stream oil, cashew shell oil, corn oil and waste engine oil) to rejuvenate aged SBSmB and found that the temperature sensitivity of the SBSmB rejuvenated with cashew shell oil was the smallest, and the rheological model analysis indicated that cashew nut shell oil prevented the binder from changing from viscous to elastic behavior during aging. The following year, they used waste oil to regenerate the aged SBSmB, and found that the waste oil mainly improved the penetration, ductility, low-temperature crack resistance, fatigue life and other characteristics of the aged SBSmB to some extent by adjusting the components of the aged bitumen [25]. However, these studies mainly used the physical rejuvenation method to supplement the light components in the aged base bitumen, so as to balance the chemical composition of the aged bitumen to a certain extent, and to achieve the purpose of partially improving the performance of the aged SBSmB [26,27,28]. Although the low-temperature performance and fatigue characteristics of aged SBSmB were significantly improved after recycling with common oil-based rejuvenators, its high-temperature stability, and viscoelastic characteristics were not effectively guaranteed [24,25,29]. The main reason is that conventional physical rejuvenation methods, such as adding aromatic oil, will mainly cause a significant deterioration in high-temperature deformation resistance, and will also bring about a decreasing adhesion of the rejuvenated binder to aggregates. However, little research can be found that has led to the development of new techniques to solve these issues. Therefore, exploring a novel approach to reach this objective and fill the current research gap is of great significance. The research on the rejuvenation of aged SBSmB is a key subject in recent years. Studies have shown that after oxidative aging, the molecular chain of SBS in asphalt binder will be broken into some small molecular weight degradation products containing oxygen-containing groups such as –OH and –COOH, which will lead to the destruction of the original physical network structure of SBS and its asphalt binder performance loss [30,31,32]. In view of this, the selection of substances that can react with the oxidative degradation products of SBS has a positive effect on the high-performance recycling of aged SBS modified bitumen.

Single-component polyurethane (PU) is a type of resin with an active isocyanate terminal group (–NCO) [33,34]. It can react with substances (including water) containing –OH without the assistance of curing agents to form polymers with excellent properties [35,36,37]. Lu et al. [38] used polyurethane modified bitumen to prepare permeable bitumen mixture, and found that it had better bonding strength, permeability, and mechanical properties than traditional permeable asphalt pavement, and solved the problem of long service life of permeable asphalt pavement. This indicates that PU modified asphalt mixture has good comprehensive performance after curing in air [38]. Therefore, PU prepolymer has excellent reaction potential to rebuild the molecular structure of aged SBS in order to improve the high-temperature properties of physically rejuvenated SBSmB.

**Figure 1:**
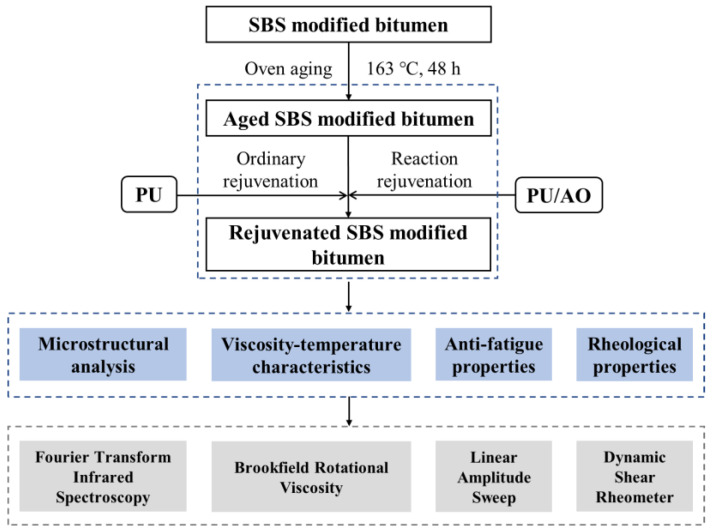
Research flowchart of this study.

In order to develop a rejuvenator that can not only rejuvenate the properties of aged bitumen but also the structure and properties of the SBS modifier, this study proposed a new physicochemical rejuvenation method based on the main reconstruction of the molecular structure of aged SBS. PU prepolymer was used as a reactive rejuvenator to react with active oxygen-containing groups, and AO was used as the bitumen component-returning agent to adjust the chemical composition of aged SBS modified bitumen. The effect of synergistic reaction rejuvenation of single-component polyurethane and aromatic oil on the structure and properties of aged SBS modified bitumen was evaluated by means of Fourier transform infrared (FTIR) spectroscopy, Brookfield rotational viscosity tests, linear amplitude sweep (LAS) tests [39] and dynamic shear rheometer (DSR) tests. The research flowchart of this study is presented in Figure 1.

## 2. Materials and Methods

### 2.1. Raw Materials

#### 2.1.1. SBS Modified Bitumen

The SBS modified bitumen (SBSmB) used in this study was a commercial product with a high temperature performance grade of PG76, and the modifier was added at approximately 4 wt%. The detailed physical properties are shown in Table 1.

#### 2.1.2. Single-Component Polyurethane (PU)

In this study, single-component polyurethane (prepolymer) was used as the repairing substance for structural reconstruction of aged SBSmB, as it is able to reconstruct the molecular structure of SBS degradation products to some extent, thereby improving the overall performance of rejuvenated SBSmB. The PU prepolymer has a special molecular structure, with terminal isocyanate groups (–NCO), and is proven to have a strong reaction activity with –OH, –COOH, etc. The general formula of the chemical reaction between PU prepolymer and alcohol, carboxylic acid or water is presented below:

R–NCO + X–OH→R–NHCOO–X

R–NCO + X–COOH→R–NHCOOOC–X→R–NHCO–X + CO_2_

2R–NCO + H_2_O→RNHCONHR + CO_2_

#### 2.1.3. Aromatic Oil (AO)

AO (aromatics/the total, ≥80%) was economically obtained from industrial markets and used as a common bitumen rejuvenator for aSBSmB to adjust the aging-induced unbalanced chemical composition of bitumen for improved workability. The relevant technical indexes of AO are: brown appearance by visual inspection, density of 1.05 g/cm^3^, and flash point exceeding 200 °C.

### 2.2. Preparation of Aged SBSmB

The aging of SBSmB was carried out in an air-circulating oven at 163 °C for 48 h. After aging, aSBSmB was obtained and tested through a series of physical and rheological tests. Some of the performance results are shown in Table 1.

### 2.3. Preparation of Rejuvenated SBSmB

To prepare the PU rejuvenated SBSmB (3PU/aSBSmB), 3 wt% PU by weight was added to and mixed with aSBSmB at 170 °C for 5 min. In addition, 10 wt% AO was added to 3PU/aSBSmB under the same preparation conditions to prepare the jointly rejuvenated SBSmB, namely 3PU/10AO/aSBSmB. For the dosage adoption, this is because 3 wt% PU can chemically consume all the SBS degradation products in the aged SBSmB binder, and 10 wt% AO can achieve an optimum viscosity-reducing effect on the aged binder in the preliminary study.

### 2.4. Test Methods

#### 2.4.1. Fourier Transform Infrared Spectroscopy (FTIR) Test

In this study, the NicoletTM iS50 FTIR instrument was used to test and analyze the molecular structure of the target bitumen samples, and to clarify whether PU prepolymer was able to react with aSBSmB. The attenuated total reflection method was used during the test, and the conditions were set as follows: the wave number range was 4000~400 cm^−1^, scanning number was 32 times, and the resolution was 4 cm^−1^. Different specimens of the same binder were tested twice for more accurate results, and the test was repeated more once if the two test results were not close.

#### 2.4.2. Brookfield Rotational Viscosity Test

In accordance with ASTM D4402, the viscosity of bitumen samples was measured using the NDJ-1C Brinell rotational viscometer for virgin, aged and rejuvenated SBSmB at different temperatures, with the No. 27 rotor and a 50 rpm speed being selected for the test. Two or more viscosity tests were conducted on different specimens of the same binder, and the average value of the closest results which met the requirements of the allowable test error was taken as the measured value. The test temperatures were selected as 135 °C, 150 °C, 160 °C, 170 °C, and 180 °C. When processing the test data, Saal formula was used to linearly fit the viscosity–temperature curves.

The Saal formula, recommended by the requirements of ASTM D2493, is an important linear expression to describe the viscosity–temperature characteristics of asphalt binder, as shown in Equation (1). According to the slope of the fitted curve, the temperature sensitivity of asphalt binder can be judged.

(1) $$\mathrm{lg}\left[\mathrm{lg}\left(\eta \times 10^{3}\right)\right]=n-m\mathrm{lg}\left(T+273.13\right)$$

where, *η* is the value of viscosity, Pa∙s; *m* is the regression coefficient, equal to the slope of the viscosity–temperature regression curve; *n* is the value of viscosity when the temperature of asphalt binder is zero; and *T* is Celsius, °C.

#### 2.4.3. Linear Amplitude Sweep (LAS) Tests

LAS is a fatigue test method based on the viscoelastic continuum damage theory. To conduct this test, the DSR device was employed with 8 mm parallel plates and a controlled gap distance at 2 mm. The test was performed following two stages, namely, frequency sweep and amplitude sweep. For the first stage, the frequency sweep test for each sample was performed at 25 °C with an applied stress of 0.1% strain over a range of 0.1~30 Hz. For the second stage, the amplitude sweep test was run at the same temperature in a strain-control mode at 10 Hz, and the loading scheme consisted of 10 s intervals of constant strain amplitude from 0.1% to 30%. The test for the same sample was repeated twice for error reduction. The performance parameter α of the material without damage is obtained from the frequency sweep test to calculate the parameter B in the fatigue equation. Combined with the amplitude sweep test results, the fatigue equation parameter A can be obtained, and the fatigue life *N_f_* can be also determined, accordingly.

The damage accumulation of the sample in the LAS test was calculated using Equation (2).

(2) $$D\left(t\right)=\sum_{i=1}^{N}{\left[\pi \gamma_{i}^{2}\left(C_{i-1}-C_{i}\right)\right]}^{\frac{\alpha }{\alpha +1}}{\left(t_{i}-t_{i-1}\right)}^{\frac{1}{1+\alpha }}$$

where, $C_{i}=\frac{{\left|G^{*}\right|}_{i}}{{\left|G^{*}\right|}_{initial}}$ is the integrity parameter; $G^{*}$ is the complex shear modulus, MPa; $\gamma_{i}$ is the applied strain at $t_{i}$ time, %; *t* is the testing time, s; and $\alpha =\frac{1}{m}$ is the rheological parameter of bitumen, where *m* is the slope of the best-fit straight line with log (storage modulus) on the vertical axis and log (applied frequency) on the horizontal axis.

For a specific time *t*, the relationship between $C_{t}$ and $D_{t}$ is as follows:

(3) $$C\left(t\right)=C_{0}-C_{1}{\left[D\left(t\right)\right]}^{C_{2}}$$

(4) $$\mathrm{lg}\left(C_{0}-C_{t}\right)=\mathrm{lg}\left(C_{1}\right)+C_{2}\cdot \mathrm{lg}\left(D_{t}\right)$$

The value of $D_{t}$ at fatigue failure is $D_{f}$, calculated as follows:

(5) $$D_{f}={\left(\frac{C_{0}-C_{peakstress}}{C_{1}}\right)}^{\frac{1}{C_{2}}}$$

where, $C_{peakstress}$ is the value of the C(t) corresponding to the peak shear stress.

The binder fatigue performance parameter *N_f_* can be calculated as follows:

(6) $$A=\frac{f{\left(D_{f}\right)}^{k}}{k{\left(\pi C_{1}C_{2}\right)}^{\alpha }}$$

(7) $$N_{f}=A{\left(\gamma_{\max}\right)}^{-B}$$

where, *f* is loading frequency, equal to 10 Hz; and $k=1+\left(1-C_{2}\right)\alpha$; B=2α; $\gamma_{\max}$ is estimated maximum strain of pavement.

#### 2.4.4. Dynamic Shear Rheometer (DSR) Tests

In this study, the rheological properties of bitumen samples were tested by MCR 702 DSR instrument, and the rheological parameters, such as complex modulus (G*), phase angle (δ), elastic modulus (G′) and viscous modulus (G″), were obtained. The test was repeated twice or more for different specimens of the sample binder to guarantee the accuracy of the results. The testing process was as follows: (1) rutting factor (G*/sin δ), for characterizing the high-temperature deformation resistance of bitumen samples, was tested using a pair of 25 mm parallel plates with a 1 mm gap at a control strain of 10%, an angular frequency of 10 rad/s, and a temperature of 76 °C; (2) fatigue failure temperature, for characterizing the fatigue resistance of bitumen samples, was determined by the temperature related to the fatigue factor (G*sin δ) at 5000 kPa, which was carried out using a pair of 8 mm parallel plates with a 1 mm gap at a control strain of 1%, an angular frequency of 10 rad/s, and a temperature range of 5~30 °C; (3) temperature response of viscoelastic behavior was tested using the above similar conditions at 6~30 °C and 30~62 °C, respectively; (4) Frequency response of viscoelastic behavior, following the above similar methods, was also tested in the frequency range of 0.628~188.1 rad/s at low temperature (6 °C), medium temperature (30 °C), and high temperature (62 °C), to study the rheological behaviors of bitumen samples under different climate environments.

## 3. Results and Discussion

### 3.1. Effect of Reaction Rejuvenation on Molecular Structure of aSBSmB

**Figure 2:**
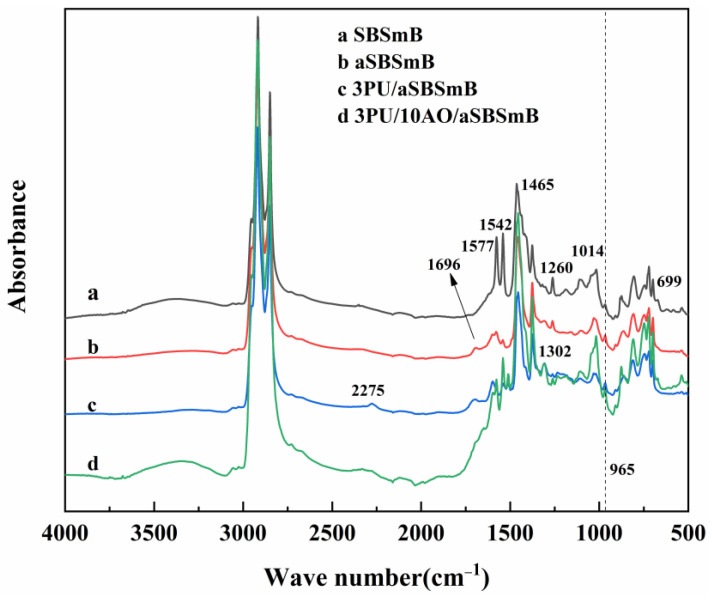
Effect of different rejuvenators on the molecular structure of aSBSmB.

FTIR spectra of SBSmB before and after aging and rejuvenation are displayed in Figure 2. In addition, the attribution of its main characteristic peaks is shown in Table 2. As seen in curves a and b, it is easy to find that, after aging, the appearance of the peaks at 1696 cm^−1^, 1014 cm^−1^ and 965 cm^−1^ are attributed to the stretching vibration of C=O, S=O and C=C, respectively. In addition, the appearance of the characteristic peak at 699 cm^−1^ was attributed to the bending vibration of C-H on the monosubstituted benzene ring. After aging, the characteristic absorption peaks at 699 cm^−1^ and 965 cm^−1^ belonging to the mono substituted benzene ring on the polystyrene segment still existed, indicating that as the thermo-oxidative aging time prolongs, the high-temperature conditions would not damage the benzene ring structure in the polystyrene segment of SBS. Meanwhile, the intensity of characteristic peaks attributed to the respiratory vibration of the benzene ring skeleton and the deformation vibration of C-H at 1260 cm^−1^ on the structure of Olefins decreased to a certain extent, indicating that aging promoted the oxidative degradation of SBS in SBSmB. When 3 wt% PU was added to aSBSmB, marked changes appeared in their corresponding spectrum. The characteristic peak at 2275 cm^−1^ is generated by the asymmetric stretching vibration of –NCO, and its intensity is obviously smaller, while the intensity of C=O is slightly enhanced. It indicates that when the rejuvenator amount of PU is 3 wt%, PU can completely react with the active oxygen-containing groups (such as –OH and –COOH) in aSBSmB, especially the oxidative degradation products of SBS. As seen in curves c and d, compared to 3PU/aSBSmB, the characteristic peak positions have no changes with the addition of AO, except for the disappearance of the characteristic peak attributed to the –NCO at 2275 cm^−1^. This shows that the chemical composition of AO is similar to that of bitumen, having a few groups that can react with PU in mixtures, which basically indicates that AO acts as a physical softening effect on the rejuvenation of aSBSmB.

### 3.2. Effect of Reaction Rejuvenation on Viscosity–Temperature Characteristic of aSBSmB

**Figure 3:**
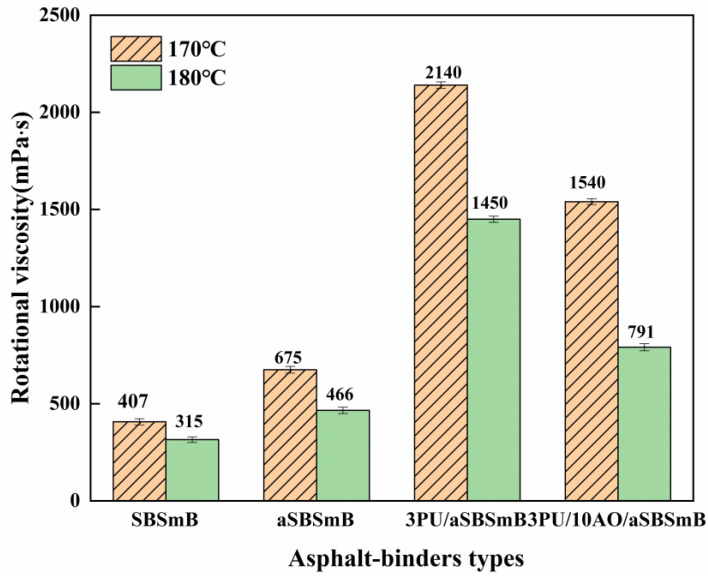
Effect of reaction rejuvenation on the viscosity of aSBSmB.

Figure 3 shows the influence of reaction rejuvenation on the viscosity of aSBSmB. It can be seen that the viscosity of SBSmB increases after aging, which indicates that the workability of SBSmB worsens after aging. The viscosity of aSBSmB at 170 °C increased significantly from 675 mPa·s to 2140 mPa·s, and even reached 1450 mPa·s at 180 °C after adding 3 wt% PU, which indicated that PU would adversely affect the workability of aSBSmB after rejuvenation. This is because PU reacts violently with the chemically active components in aSBSmB, forming a structural reinforcement with weakened high-temperature sensitivity. When 10 wt% AO was added to 3PU/aSBSmB, the viscosity of 3PU/aSBSmB decreased from 2140 mPa·s to 1540 mPa·s and from 1450 mPa·s to 791 mPa·s at 170 °C and 180 °C, respectively. The results are due to the fact that 10 wt% AO as rejuvenator can compensate for the reduction of light components by aging volatilization or reaction in aSBSmB. In addition, the dilution and dissolution of AO reduces the viscosity and hardness of 3PU/aSBSmB. From this result, it is believed that the combined use of PU and AO contributes to better workability of the rejuvenated binder for mixing and construction. It is worthwhile noting that at both 170 °C and 180 °C, the viscosity of the rejuvenated binder is still higher than that of the fresh binder, but this still satisfies the mixing and compaction requirements of the asphalt mixture.

**Figure 4:**
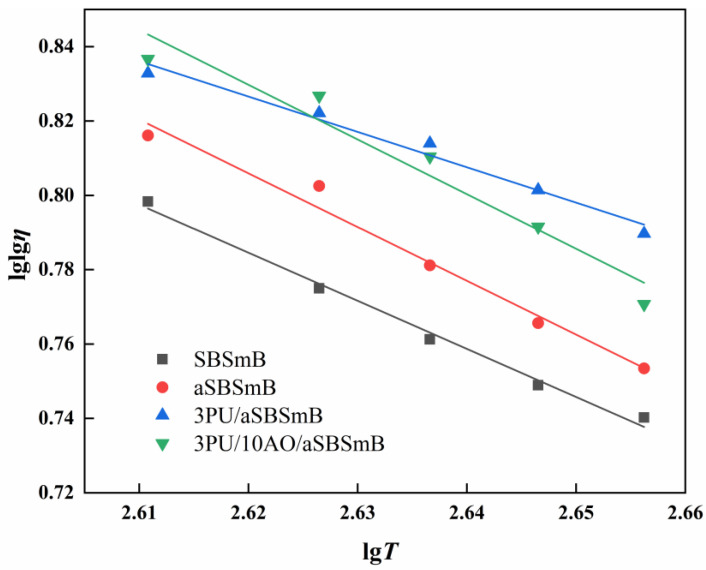
Effect of reaction rejuvenation on the viscosity–temperature characteristic of aSBSmB.

Figure 4 shows the viscosity–temperature regression curves of virgin, aged and rejuvenated SBSmB, of which the linear fitting equations, regression coefficients and correlation coefficients are shown in Table 3. The correlation coefficients R^2^ of the viscosity–temperature curves after linear fitting are not less than 0.95, indicating that the linear fitting curves can truly reflect the relationship between viscosity and temperature of virgin, aged and rejuvenated SBS modified bitumen. When SBSmB is aged, the regression coefficient (the value of m) increases from 1.293 to 1.446, indicating that the viscosity–temperature sensitivity of SBSmB enhance significantly after aging. This is mainly due to the loss of light components in the aSBSmB, resulting in a larger molecular weight of the bitumen matrix and a harder texture of the SBS modified asphalt binder. With the addition of 3 wt% of PU as rejuvenator, the value of m of the reaction-rejuvenated SBS modified asphalt binder decreased to 0.951, indicating that the addition of PU will reduce the viscosity–temperature sensitivity. This is because PU reacts violently with the chemically active components in aSBSmB to make the molecules rotate relatively and does not harden the SBSmB after structural reconstruction, so as to reduce the viscosity–temperature sensitivity. After 10 wt% AO was added to the above 3PU/aSBSmB, it was found that the value of m is restored to 1.459, indicating that the addition of AO can enhance the viscosity–temperature sensitivity of 3PU/aSBSmB for better workability.

### 3.3. Anti-Fatigue Properties of Rejuvenated SBSmB

#### 3.3.1. Variation of Integrity Parameter (*C*) with Cumulative Fatigue Damage Parameter (*D*)

**Figure 5:**
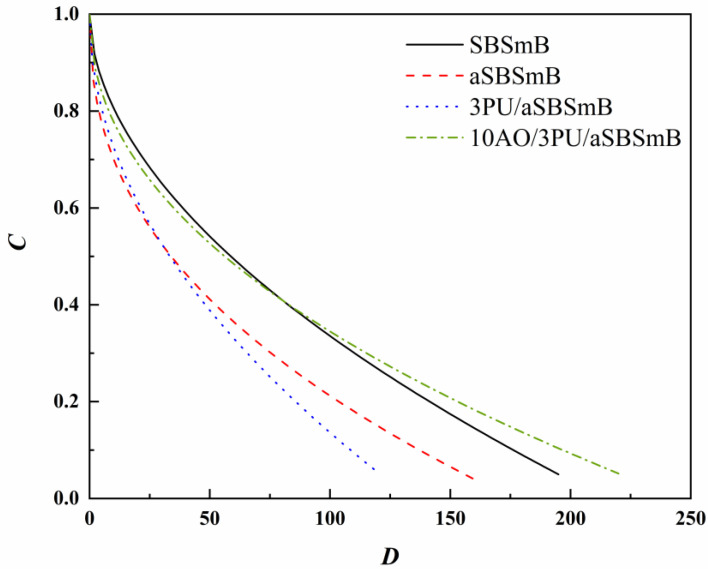
*C*-*D* curves of the virgin, aged, and rejuvenated SBSmB.

The initial integrity (*C*) without damage is determined as a value of 1, and 0 represents the complete damage of the binder. For a specific *D* value, the lower the *C* value, the higher the damage degree of bitumen, and the worse the fatigue damage resistance of bitumen. Figure 5 shows the relationship between the integrity parameter (*C*) and cumulative fatigue damage parameter (*D*) of the virgin, aged, and rejuvenated SBSmB. After aging, the integrity curve of SBSmB becomes steeper, which indicates that the loss rate is significantly increased, and the fatigue resistance is worse than that of virgin SBSmB. When 3 wt% PU was added, the fatigue resistance performance of aSBSmB was visually reduced. This may be due to the negative influence of PU on the colloidal structure of aged SBSmB. By contrast, the combined use of PU and AO promotes not only the rejuvenation of network structure, but also the restoration of bitumen fractions in aged SBSmB, leading to the better resistance of 3PU/10AO/aSBSmB to fatigue.

#### 3.3.2. Number of Cycles to Fatigue Failure (*N_f_*)

**Figure 6:**
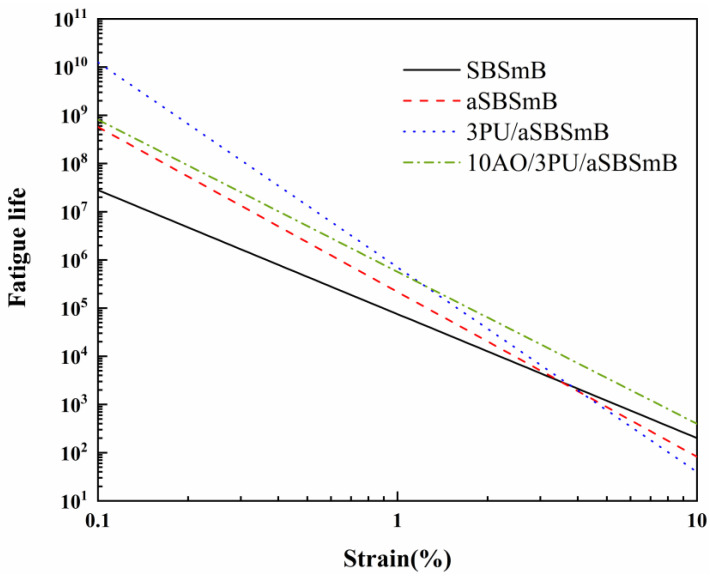
Fatigue life prediction for the virgin, aged, and rejuvenated SBSmB.

The numbers of cycles at fatigue failure of SBSmB before and after aging and after rejuvenation is shown in Figure 6 and Table 4. It is clear that the curve slope of aSBSmB is bigger than that of the fresh binder, indicating that the fatigue life of aSBSmB is by contrast more sensitive to load. As PU precursor plays an important role in the rejuvenation, its rejuvenated binder shows a higher fatigue life compared to aged one at a smaller strain, such as below 1%, because of its reaction-enhancing effects from the chemical rejuvenation of degradation products. In addition, *N_f_* of 3PU/10AO/aSBSmB presents a higher value than that of 3PU/aSBSmB at higher strain of over 1%, which further demonstrates that AO can help significantly improve the fatigue resistance of PU rejuvenated binder at high strains. Overall, the combined use of PU and AO can positively contribute to enhancing the fatigue properties of aged binder, particularly at higher stains.

### 3.4. Evaluation of Anti-Deformation and Fatigue Performance of Reaction Rejuvenation SBSmB

**Figure 7:**
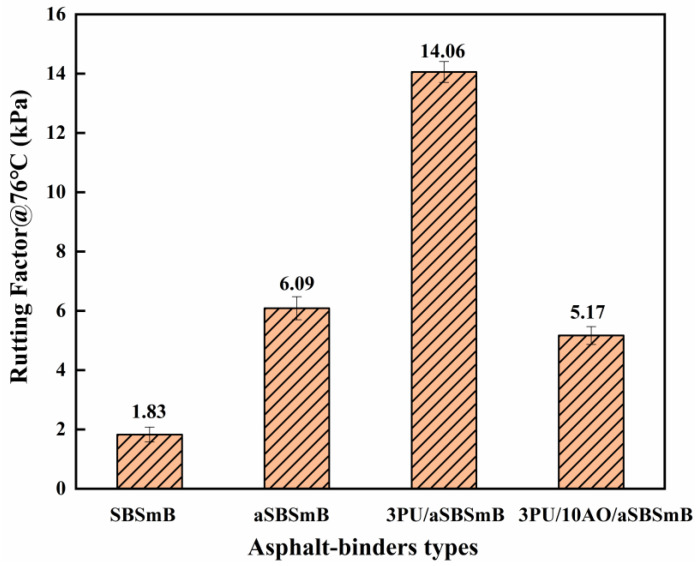
Effect of reaction rejuvenation on the rutting deformation resistance of aSBSmB.

**Figure 8:**
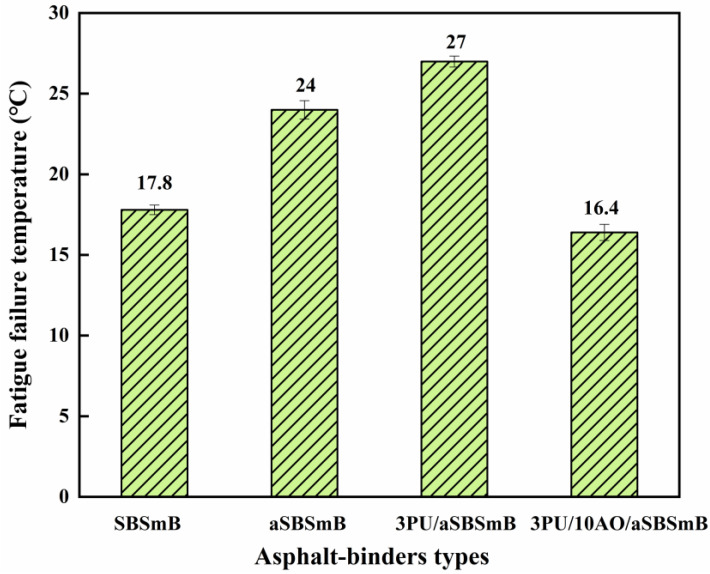
Effect of reaction rejuvenation on the fatigue failure temperature of aSBSmB.

Figure 7 and Figure 8, respectively, reflect the influence of reaction rejuvenation on anti-rutting deformation and the anti-fatigue characteristics of aSBSmB. It can be seen that the 76 °C rutting factor and fatigue failure temperature of SBSmB after aging both increase significantly, from 1.83 kPa to 6.09 kPa and from 17.8 °C to 24.0 °C, respectively, which indicates that the deformation resistance of SBSmB can be improved after aging, but its fatigue property is prone to failure at higher temperatures. With the addition of 3 wt% PU, the rutting factor of aSBSmB at 76 °C increased significantly from 6.09 kPa to 14.06 kPa, with an increase of 130.9%, and its fatigue failure temperature increased to 27.0 °C, indicating that PU can significantly improve the deformation resistance of aSBSmB, but it will also reduce its fatigue resistance to some extent. Therefore, when 10 wt% AO is added to 3PU/aSBSmB, the rutting factor of rejuvenated SBSmB at 76 °C can still be kept at 5.17 kPa, and its fatigue failure temperature (16.4 °C) is lower than that of the virgin SBSmB (17.8 °C), which indicates that the synergistic rejuvenation effect of PU and AO can not only ensure the high-temperature stability of rejuvenated SBSmB, but can also promote its ability to resist fatigue damage. This is because the chemical reaction between PU and SBS oxidation degradation products dominates the high-temperature characteristics of rejuvenated SBSmB, while AO, as a softening agent of hard components in aged bitumen, supplements the light components, which positively affects the fatigue performance of rejuvenated SBSmB.

### 3.5. Temperature Response of Viscoelastic Behavior of Reaction Rejuvenation SBSmB

**Figure 9:**
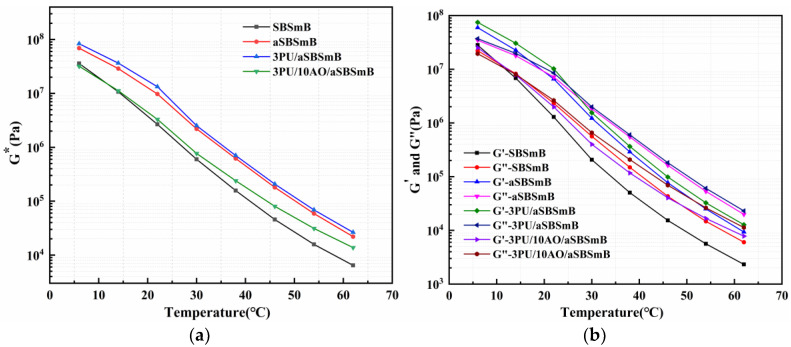
Temperature response to viscoelastic behavior of reaction-rejuvenated SBSmB: (**a**) Complex Modulus; (**b**) Elastic/Viscous Modulus.

Figure 9 shows the temperature response of the viscoelastic behavior of SBSmB. It can be seen from Figure 9a that after aging, the complex modulus of SBSmB obviously increases within the design temperature range. When 3 wt% PU was added to aSBSmB, its complex modulus increased slightly, but after adding 10 wt% AO, the complex modulus of rejuvenated SBSmB was slightly higher than that of virgin SBSmB. These results showed that the synergistic reaction and rejuvenation of PU and AO can make the deformation resistance of aged SBSmB slightly higher than that of the virgin SBSmB. The reason for this result is consistent with the analysis of the performance results obtained in Section 3.3 and Section 3.4 above. In addition, from Figure 9b, the elastic modulus G′ or viscous modulus G″ of SBSmB before and after aging and rejuvenation is consistent with the change law of its corresponding complex modulus, except that its viscoelastic transition temperature (that is, the temperature corresponding to the intersection of G′-T and G″-T curve) is different. It showed that the viscoelastic transition temperature of SBSmB increases from 11.8 °C to 19.9 °C after aging. Further, 3 wt% PU promoted the viscoelastic transition temperature of aSBSmB to continue to increase to 25.2 °C. Then, when 10 wt% AO was added to 3PU/aSBSmB, this value decreased to 12.4 °C, and basically returned to the virgin SBSmB level. These results indicate that the interaction between PU and aSBSmB will lead to the elastic behavior of PU rejuvenated SBSmB, and the elastic behavior of rejuvenated SBSmB will gradually develop to viscous behavior after the softening action of appropriate amount of AO, showing the viscoelastic behavior characteristics close to the virgin SBSmB. The main reason for this result is the chemical interaction between PU and oxidation degradation products of SBS after aging, which organically reconstructs the molecular structure of the modifier and forms the crosslinked structure with relatively difficult molecular movement. Then, through the permeation plasticization of AO, the intermolecular force of aging asphalt is reduced, and the rejuvenated SBSmB with stable physical and chemical structure is achieved.

### 3.6. Frequency Response of Viscoelastic Behavior of Rejuvenated SBSmB at Different Temperatures

**Figure 10:**
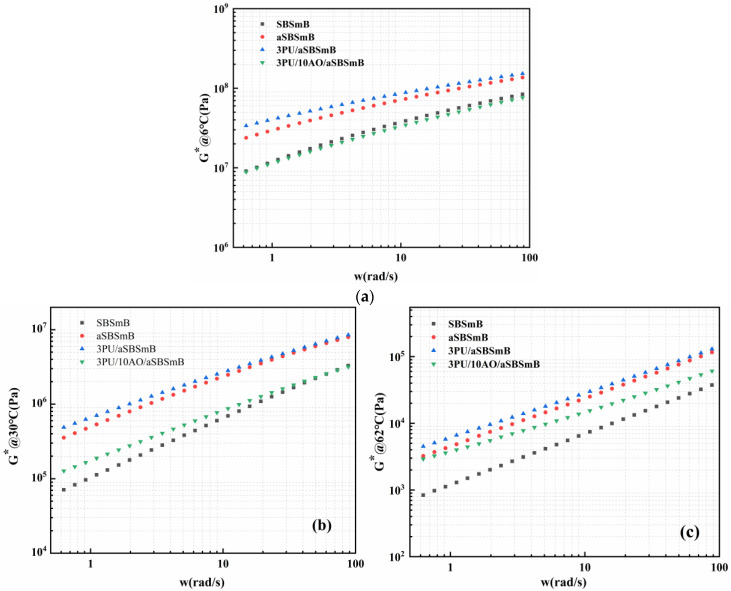
Frequency response to viscoelastic behavior of reaction-rejuvenated SBSmB at various temperatures: (**a**) low temperature, 6 °C; (**b**) medium temperature, 30 °C; (**c**) high temperature, 62 °C.

Figure 10 shows the frequency response of viscoelastic behavior of SBSmB at different temperatures. It can be seen that the addition of 3 wt% PU will increase the complex modulus of aSBSmB in the set frequency range (0.628~88.1 rad/s) regardless of the ambient temperature. However, after adding 10 wt% AO continuously, the frequency response of the complex modulus of rejuvenated SBSmB at different temperatures is different, specifically, its viscoelastic behavior is very close to the level of the virgin SBSmB at low temperature; when the temperature transits from low temperature to medium temperature, its viscoelastic behavior, which is characterized by elasticity, gradually approaches the level of the virgin SBSmB with the increase of frequency. At high temperature, its elastic resistance behavior is dominant, which is higher than that of the virgin SBSmB. These results show that the frequency response of SBSmB rejuvenated with an appropriate amount of PU and AO is similar to that of the virgin SBSmB in a low-temperature environment, but it mainly shows a stronger frequency response than that of the virgin SBSmB in medium- and high-temperature environments. This is because, at low temperature, the movement of small molecule AO provides internal plasticization for the stable structure rebuilt by PU and the aged bitumen molecules, which makes the rejuvenated SBSmB obtain the viscoelastic characteristics equivalent to the virgin SBSmB. At medium and high temperature, although the movement of AO molecules intensifies, the rejuvenated crosslinked structure of SBS degradation products rebuilt by PU shows stable high-temperature characteristics.

**Figure 11:**
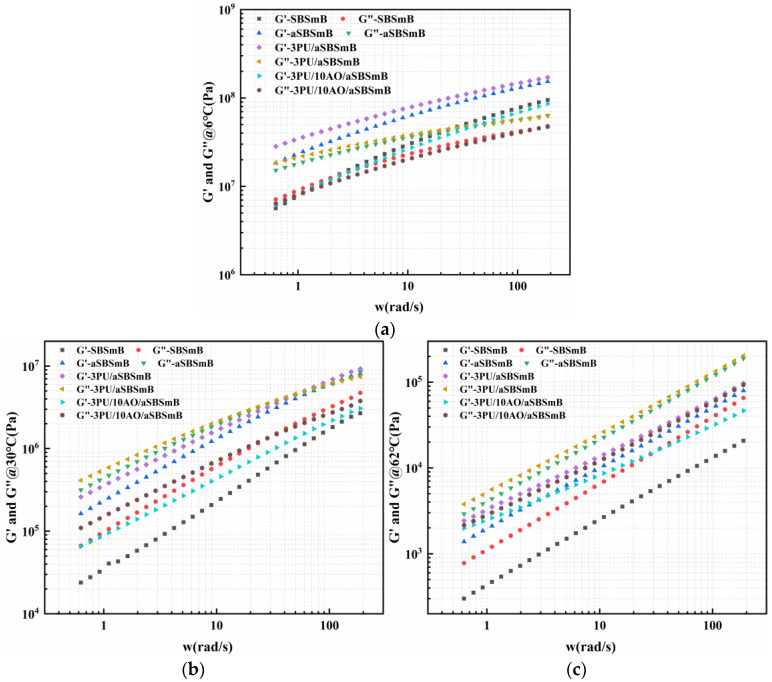
Frequency response to viscoelastic transition of reaction-rejuvenated SBSmB at various temperatures: (**a**) low temperature, 6 °C; (**b**) medium temperature, 30 °C; (**c**) high temperature, 62 °C.

To further clarify the above viewpoint, the frequency response of viscoelastic transition of reaction-rejuvenated SBSmB is shown in Figure 11. It can be seen that with the increasing frequency, the virgin SBSmB has a tendency to change from viscous behavior to elastic behavior at about 2.5 rad/s at low temperature, and other binders present a tendency of elastic behavior in the whole frequency and temperature range. In addition, at low temperature, the elastic modulus and viscous modulus of the rejuvenated SBSmB are close to those of the virgin SBSmB, while at medium and high temperature, the elastic modulus and viscous modulus of the rejuvenated SBSmB are both higher than that of virgin SBSmB. It indicates that compared to virgin SBSmB, the rejuvenated SBSmB basically has the similar viscoelastic behavior at low temperature, and it has better ability to resist elastic deformation at medium and high temperature. The reason for this result is consistent with the above analysis.

## 4. Conclusions

(1) This study proposed a new physicochemical rejuvenation method based on the novel idea of rebuilding the molecular structure of aged SBS. PU prepolymer was used as a reactive substance to react with active oxygen-containing groups from the degradation products of SBS, and AO was used as a common rejuvenator to supplement the lost light fractions of asphalt molecules in aged SBSmB. With respect to the structure and performance characterizations, a series of tests, including infrared spectra, viscosity, and dynamic shear rheology, were carried out for the rejuvenation analysis of aged SBSmB. Based on this, the main conclusions can be drawn: FTIR results indicated that PU can react with oxidative degradation products of SBS, such as -OH and -COOH, for structural reconstruction, while AO can soften the aged virgin bitumen through the reduction of heavy fractions.
(2) RV results suggested that the increased viscosity of aged SBSmB after adding PU can be effectively decreased to a better workability with a certain incorporation of PU.
(3) LAS results demonstrated that the combined use of PU and AO can reach a better resistance of aged SBSmB to fatigue by contrast to single use of PU, because of chemical rejuvenation of the network structure of aged SBS and the physical softening rejuvenation of aged bitumen components.
(4) Combined results of rutting and fatigue factors stated that the chemical reaction between PU and SBS degradation products dominates the high-temperature stability of rejuvenated SBSmB, while AO supplements the light components in aged bitumen and improves the fatigue performance of rejuvenated SBSmB.
(5) Viscoelasticity results identify that, compared to virgin SBSmB, PU/AO rejuvenated SBSmB has a similar viscoelastic behavior at low temperature, while it also has better resistance to elastic deformation at medium and high temperatures.

Overall, it is suggested to add an appropriate amount of PU prepolymer to the physically rejuvenated SBSmB binder to maintain better rheological properties and deformation resistance, for the purpose of high-quality recycling and application of waste SBS modified asphalt mixtures. Future works should include not only evaluation of mixture properties, but also benefit analyses of cost, environmental, and social benefits.

## Figures and Tables

**Table 1:** Basic physical properties of SBS modified bitumen before and after aging.

Parameters	Measured Results		Technical Criterion	Specifications
	SBSmB	aSBSmB		
Softening point (°C)	63.8	72.4	>60	ASTM D36
Penetration at 25 °C, 100 g, 5 s (0.1 mm)	58	46	40~60	ASTM D5
Viscosity at 135 °C (mPa·s)	1930	3530	≤3000	AASHTO TP48
Viscosity at 150 °C (mPa·s)	904	2220	≤3000	AASHTO TP48
Rutting factor at 76 °C (kPa)	1.83	6.09	>1.0 kPa	ASTM D6373

**Table 2:** Attributes of the main characteristic peaks.

Wave Number (cm^−1^)	Attribution	Vibration Type
2275	-NCO	-N=C=O Asymmetric Stretching
1696	C=O	C=O Stretching
1577, 1542, 1465	Benzene ring	Respiratory skeleton
1302	C-NH_2_ or C-NH-	C-N Stretching
1260	Olefin	C-H Bending
1014	S=O	S=O Stretching
965, 699	-CH_2_=CH_2_-	C-H Bending

**Table 3:** Viscosity–temperature curve regression analysis.

Types of Binder	Viscosity–Temperature Equations	*m*	R^2^
SBSmB	$\mathrm{lglg}\left(\eta \times 10^{3}\right)=4.173-1.293\mathrm{lg}\left(T+273.13\right)$	1.293	0.992
aSBSmB	$\mathrm{lglg}\left(\eta \times 10^{3}\right)=4.595-1.446\mathrm{lg}\left(T+273.13\right)$	1.446	0.981
3PU/aSBSmB	$\mathrm{lglg}\left(\eta \times 10^{3}\right)=3.318-0.951\mathrm{lg}\left(T+273.13\right)$	0.951	0.978
3PU/10AO/aSBSmB	$\mathrm{lglg}\left(\eta \times 10^{3}\right)=4.683-1.459\mathrm{lg}\left(T+273.13\right)$	1.459	0.950

**Table 4:** Fatigue life comparison of target binders.

Binder Type	Equations Related to the Fatigue Curves
SBSmB	$N_{f}=75,069.25\gamma_{\max}^{-2.58}$
aSBSmB	$N_{f}=217,091.60\gamma_{\max}^{-3.42}$
3PU/aSBSmB	$N_{f}=706,365.02\gamma_{\max}^{-4.25}$
3PU/10AO/aSBSmB	$N_{f}=567,672.03\gamma_{\max}^{-3.16}$

## Data Availability

Not applicable.
